# Immune-reactive tumor organoids system to determine the effects of microbial metabolites on cancer immunity and immunotherapies

**DOI:** 10.3389/frmbi.2024.1411322

**Published:** 2024-10-07

**Authors:** Azza M. El-Derby, Cecilia R. Schaaf, Ethan Shelkey, Katherine L. Cook, Konstantinos I. Votanopoulos, Shay Soker

**Affiliations:** ^1^ Wake Forest Institute for Regenerative Medicine, Wake Forest University School of Medicine, Winston Salem, NC, United States; ^2^ Pathology section, Comparative Medicine, Wake Forest School of Medicine, Winston Salem, NC, United States; ^3^ Department of Cancer Biology, Wake Forest School of Medicine, Winston Salem, NC, United States; ^4^ Hypertension and Vascular Research Center, Wake Forest University School of Medicine, Winston Salem, NC, United States; ^5^ Division of Surgical Oncology, Wake Forest Baptist Health, Winston Salem, NC, United States

**Keywords:** cancer, immunotherapy, *in vitro*, microfluidics, hydrogels, organoid

## Abstract

Immunotherapies are a revolutionary approach to treating cancer by utilizing the body’s immune system to target and combat cancer cells. This approach offers promising alternatives to traditional chemotherapies. Its potential to induce long-lasting remissions and specificity for cancer cells, which minimizes side effects, makes it a cutting-edge treatment with tremendous potential. With the increase of the clinical usage of immunotherapy, evidence emerges of the microbiome’s impact on both tumor growth and response to immunotherapy. The proposed involvement of the microbiome can change treatment efficacy by altering drug metabolism and reshaping the immune system response. Understanding the specific interactions between tumor cells, immune cells, and the microbiome is a critical step in the advancement of immunotherapy. To study the complex interaction between cancer immunity and the microbiome, various preclinical *in vivo* and *in vitro* models have been developed. We have recently described the use of an *ex vivo* preclinical model for anti-cancer treatment outcome prediction –tumor tissue equivalents (organoids). Specifically, immune-reactive tumor organoids are proposed as a novel tool for understanding how the microbiome influences cancer immunity and immunotherapy. More importantly, this platform can utilize patient samples to dissect patient-specific elements regulating cancer immune response and microbiome influence. This review presents the rationale for using the immune-reactive tumor organoids model to study the interactions between the microbiome and cancer immunotherapy. It will discuss available components of the model and analyze their interplay, summarize relevant experimental data, and assess their validity. Additionally, it explores the potential of immune-reactive organoids for personalized treatment approaches. Understanding the microbiome’s role in immunotherapy outcomes will lead to transformative cancer treatment via a simple change of diet or other microbiome manipulations. Ongoing research on microbiome-cancer interactions utilizing the described model systems will lead to innovative treatment strategies and improved patient outcomes.

## 1 Cancer immunology and immune therapy

The foundations of tumor immunology were established by Lloyd J. Old when he described the concepts of immunosurveillance and identified both tumor antigens and escape mechanisms (Old, 1981; Old, 1985; Kaplan et al., 1998). They demonstrated that tumors could be rejected by transferring immune cells from immunized mice into naive mice providing early evidence for cancer immunotherapy using immune cells (Old et al., 1962). These and other early discoveries paved the way to a broad array of cancer treatments that could be classified as cancer immunotherapies. Cancer immunotherapy, put simply, is a type of treatment that harnesses the power of the immune system to recognize and attack cancer cells. There are several approaches that cancer immunotherapy can utilize, and these therapies have revolutionized cancer treatment in since their introduction (Kotla et al., 2009; Kronke et al., 2015). Currently, the two main clinical therapies are checkpoint inhibitors and adoptive cell therapy (ACT).

Checkpoint inhibitors are drugs that obstruct specific proteins on immune cells or cancer cells to prevent the suppression of immune responses against tumors. Current clinically approved targets for inhibitory checkpoints include CTLA-4 and PD-1. Researchers have found that by blocking these inhibitory checkpoint proteins, immune checkpoint inhibitor antibodies effectively take the brakes off the immune system, amplifying its cancer-fighting abilities (Esfahani et al., 2020).

Adoptive cellular therapy is based on the activation and manipulation of autologous T cells in an *in vitro* setting followed by reinfusion to achieve better tumor targeting. This modality has three main approaches: tumor-infiltrating lymphocyte therapy (TILs), T cell receptor (TCR) gene therapy, and chimeric antigen receptor (CAR) modified T cells (June et al., 2015).

Tumor infiltrating lymphocyte (TILs) therapy involves expanding autologous TILs from resected tumors *ex vivo* and infusing them back into patients after lymphodepletion. In animal models, TILs from various mouse tumors have demonstrated the ability to combat tumors *in vivo* (Forget et al., 2018). Human patient studies by Radvanyi et al. showed TILs from melanomas reliably recognize autologous tumors (Radvanyi et al., 2012). In various phase I/II clinical trials for metastatic melanoma, TIL therapy resulted in tumor responses nearing 50% (Andersen et al., 2016). As of early 2024, the Food and Drug Administration approved Lifileucel from Iovance Biotherapeutics (Amtagvi) as the first approved solid tumor derived autologous T cell immunotherapy.

T cell receptor gene therapy (TCR) serves as another avenue for ACT, where patient T cells are reprogrammed to identify tumor antigens by introducing genes that encode tumor-specific TCRs (D’Angelo et al., 2018). One of the notable targets in this therapy includes cancer germline antigens such as NY-ESO-1. NY-ESO-1 is expressed in up to 52% of melanomas (Goydos et al., 2001), neuroblastomas (Camisaschi et al., 2018), synovial sarcomas and mixoid and round cell liposarcomas, and ovarian cancer (Robbins et al., 2015). Research has shown promising results, with response rates reaching up to 30% in melanoma cases (Robbins et al., 2015). However, there are concerns that toxicities might arise from off-tumor reactivity.

Chimeric Antigen Receptor T-cell (CAR-T) therapy is a prominent form of adoptive cell transfer (ACT), using engineered T cells to target tumor antigens through chimeric antigen receptors. These chimeric receptors incorporate parts of various other receptors to optimize cellular function. The CAR-T cells have achieved remarkable success targeting CD19 in blood cancers, with high response rates in lymphoma (Davila et al., 2014; Miller and Maus, 2015). However, translating CAR-T cell efficacy to solid tumors poses numerous challenges like the tumor immunosuppressive microenvironment and extensive extracellular matrix surrounds solid tumors, often physically blocking CAR-T migration and tumor penetration (Martinez and Moon, 2019; Donnadieu et al., 2020).

Overall, immunomodulatory drugs offer diverse approaches within cancer immunotherapy. Often used in combinations to maximize effectiveness, immunotherapeutic regimen design depends on the type of cancer, the stage, and the patient’s overall health (Wang et al., 2022). Additionally, emerging research suggests that a patient’s microbiome can significantly influence the effectiveness of cancer immunotherapy, leading to the development of therapies aimed to manipulate the microbiome and enhance the immune response against cancer.

## 2 The influence of the microbiome on cancer immune therapy effectiveness

The human microbiome, comprising trillions of microorganisms, has gained attention due to its pivotal role in health and disease, particularly its potential influence on cancer and cancer immunotherapy. The microbiome’s composition and diversity can significantly influence immune responses, making it an intriguing factor to investigate in the context of personalized cancer treatment (Gopalakrishnan et al., 2018a).

The intersections between the microbiome and cancer can be categorized at two levels (Jain et al., 2021). The first is between microbiome composition and the development of tumor, and the second between the microbiome and the immune system which in turn can direct the immune response to cancer immune therapy. The impact of microbes on the immune response and their potential as therapeutic targets in cancer treatment have been subjects of interest in recent research (Barukčić, 2017; Turna et al., 2017; Priya et al., 2022).

Metabolites and cytokines are one of the main methods of crosstalk between the microbiome and the immune system. The gut microbiota produces various metabolites and cytokines that can shape systemic immunity. For example, certain bacteria metabolize dietary fiber into short-chain fatty acids (SCFA) like butyrate, which have anti-inflammatory effects and promote T-cell differentiation (Maslowski et al., 2009; Kim et al., 2014). Gut microbe released SCFAs enter circulation and modulate systemic immune cell differentiation and function. SCFAs can promote regulatory and T helper cell 1 and 17 cell differentiation and hence affect the immune therapy activity (Kim et al., 2016). Also, the bacterium *B. pseudolongum* produces inosine which enhances checkpoint inhibitor efficacy (Mager et al., 2020). This effect could be modulated through the inhibition of ubiquitin-activating enzyme UBA6 in tumor cells which in turn augment its sensitivity to the cytotoxic activity of T cells (Zhang et al., 2022). Interestingly, some metabolites like butyrate can also have concentration-dependent opposite effects such that low levels induce T regulatory cells while higher levels boost CD8+ T cell effector activity. In the clinical setting, responsive cancer patients were reported to show a higher systemic SCFA level (Arpaia et al., 2013).

Several strategies, including fecal microbiota transplantation (FMT) and probiotic administration, have thus been adopted in attempts to enhance cancer immunotherapy efficacy. In a study by the Vétizou group (Mao et al., 2021) examining both mouse models and melanoma patient samples, it was revealed that the therapeutic efficacy of CTLA-4 blockade is dependent on key gut commensals such as *Bacteroides thetaiotaomicron* and *B. fragilis*. Furthermore, the presence of T cells specific for these bacteria was associated with superior CTLA-4 response rates. Depletion of these bacterial populations, either in germ-free mice or through antibiotic treatment, mitigated the therapeutic effect. However, repopulation using probiotics or T-cell-targeted approaches could restore responsiveness to CTLA-4 inhibition.

Along the lines of microbiota manipulation to enhance the cancer immunotherapy response, another study identified that *Bifidobacterium* administration, either through fecal transfer or oral routes, was able to halt melanoma progression similarly to PD-L1 antibody therapy. Furthermore, the combined effects of *Bifidobacterium* and PD-L1 blockade together led to a significant inhibition of tumor growth. This highlights the synergistic potential of microbial secretants and checkpoint inhibitors in overcoming cancer progression (Sivan et al., 2015). Another preclinical study in germ-free mice showed that FMT from responder patients can replicate patient response to checkpoint inhibitors; non-responder phenotype in mice is reversible with additional FMT from responders (Routy et al., 2018). Several clinical trials are now testing microbiome restoration to improve immunotherapy response. For example, in melanoma patients refractory to anti-PD-1 treatment, FMT was tested in combination with pembrolizumab. This combined therapy resulted in stabilized disease or tumor regression in 2 patients out of 3 [clinical trial: NCT 03341143]. Given these promising results, the mechanism behind the gut microbiome modulating the cancer immune therapy response is of great interest to optimize and direct the use of microbiome manipulation, either at the study design level or for therapeutic intervention.

One of the commonly reported mechanisms for microbiome interaction with immunotherapy response is through the presentation of pro-inflammatory antigens. The gut microbiome interacts with host immune cells via pattern recognition receptors like Toll-like receptors, stimulating inflammatory responses that help shape tumor immunity (Sato and Kawakami, 2022). Gut bacteria provide antigens that are sampled by intestinal immune cells and transported to lymphoid tissues to initiate B cell and T cell responses. Cross-reactivity between microbial and tumor antigens can boost anti-tumor immunity not only locally but systematically (Mowat and Agace, 2014).

A study highlighted the concept of systemic circulation of bacterial antigens and their role in cross-reactivity with pancreatic tumor antigens (Daillère et al., 2016). It reported that translocation of *Enterococcus hirae* from the gut to lymph nodes/spleen during cyclophosphamide chemotherapy enhanced anti-tumor immune responses in mice. This effect was linked to an *E. hirae* bacteriophage antigen cross-reactive with a tumor antigen, highlighting microbiome antigen mimicry of tumor antigens as an immunomodulatory mechanism (Fluckiger et al., 2020).

### 2.1 Clinical evidence of association between the microbiome and cancer immunotherapy outcomes

Multiple clinical trials across diverse cancer types like melanoma, lung, renal, gastrointestinal, thoracic, and hepatobiliary cancers have explored associations between the gut microbiome composition and outcomes with immuno-oncology treatments, especially immune checkpoint inhibitors (ICIs) like anti-PD-1 therapy (Gopalakrishnan et al., 2018b; Routy et al., 2018; Jin et al., 2019; Baruch et al., 2021; Mao et al., 2021; Yin et al., 2021; Che et al., 2022; Derosa et al., 2022; Lee et al., 2022; Pietrzak et al., 2022). Common techniques utilized included 16S rRNA sequencing, metagenomics shotgun sequencing, and metabolomics profiling of longitudinally collected fecal samples before and during ICI treatment to characterize taxonomic and functional changes. Key findings across several studies suggested that intrinsic microbiome diversity and richness are strongly associated with improved rates of clinical response to ICIs including progression-free survival, and overall survival (Jin et al., 2019; Baruch et al., 2021; Derosa et al., 2022; Pietrzak et al., 2022). Specific taxa enriched in responder groups included *Akkermansia muciniphila*, *Bifidobacterium pseudocatenulatum*, *Prevotella* spp., *Ruminococcaceae* spp., and *Bacteroides* while non-responders had increased abundance of *Proteobacteria* and *Firmicutes* species (Zheng et al., 2019; Peng et al., 2020) (Baruch et al., 2021; Lee et al., 2022; Pietrzak et al., 2022; Wu et al., 2022). Though no microbiome biomarkers could consistently differentiate response groups across diverse cohorts, these bacteria could modulate antitumor immunity via metabolite production or immune stimulation including tryptophan-derived metabolite indole-3-propionic acid (IPA) that was shown to enhance the efficacy of CD8+ T cell-mediated αPD-1 immunotherapy and via immune stimulation (Yin et al., 2021; Lee et al., 2022). Interventions like fecal microbiota transplantation from responders to germ-free mice or patients heightened ICI efficacy by favorably restoring gut homeostasis (Routy et al., 2018; Baruch et al., 2021). These studies are summarized in **Table 1**. It describes the cancer type, therapy used, microbiome intervention, antibiotic use, readouts, outcomes, and details for the trials.

**Table 1:** Clinical studies for microbiome and immunotherapy efficacy in cancer patients.

Tumor Type	Number of cases	Procedure	Immuno-therapy	Outcomes	Ref
Melanoma	112 of responder and non-responder patients	Examined oral and gut microbiome of patients undergoing anti-PD-1 immunotherapy; Analysis of patient fecal microbiome samples; metagenomic studies	Anti PD-1	Significantly higher alpha diversity and relative abundance of *Ruminococcaceae* bacteria in responding patients; Functional differences in gut bacteria including enrichment of anabolic pathways; Enhanced systemic and antitumor immunity in responding patients and germ-free mice receiving fecal transplants from responding patients	(Gopalakrishnan et al., 2018b)
Advanced NSCLC, RCC, urothelial carcinoma	Advanced NSCLC (n = 140), RCC (n = 67), or urothelial carcinoma (n = 42)	Fecal microbiota transplantation (FMT) from cancer patients to mice; examination of gut microbiome’s impact on ICIs; metagenomics of patient stool samples; analysis of gut microbiome impact on PD-1 blockade	Anti–PD-1/PD-L1	Abnormal gut microbiome composition linked to primary resistance to ICIs; correlation with the relative abundance of *Akkermansia muciniphila*; Oral supplementation with *A. muciniphila* after FMT with nonresponder feces restored efficacy of PD-1 blockade via recruitment of specific T lymphocyte subsets; Antibiotics inhibited ICIs’ clinical benefit;	(Routy et al., 2018)
Hepatocellular carcinoma (HCC)	8	Metagenomic sequencing of fecal samples; comparison of microbiome between responders and non-responders to anti-PD-1 therapy	Anti PD-1	Higher taxa richness and gene counts in responder fecal samples; Increased dissimilarity in beta diversity distinguishable at 6 weeks; Increase of Proteobacteria at 3 weeks in non-responders; 20 species enriched in responders including Akkermansia muciniphila; Functional analysis verified potential bioactivities of responder species	(Zheng et al., 2019)
Advanced non-small cell lung cancer (NSCLC)	37	Fecal sample collection at key treatment milestones; 16S ribosome RNA gene sequencing for gut microbiota profiling; peripheral immune signatures assessed by multicolor flow cytometry; Study designed to explore relationship between gut microbiome and outcomes with anti-PD-1 treatment in East Asian NSCLC population	Anti PD-1	Patients with higher gut microbiome diversity at baseline had better clinical responses to anti-PD-1 immunotherapy (were responders) compared to those with lower diversity (nonresponders); Responders had higher abundance of certain gut microbes (*Alistipes putredinis, Bifidobacterium longum*, and *Prevotella copri*) while nonresponders had higher levels of *Ruminococcus* unclassified; strong correlation between high microbiome diversity and favorable immune responses to anti-PD-1 therapy in Chinese NSCLC patients	(Jin et al., 2019)
Gastrointestinal (GI) Cancer	74 patients	Study Association of gut microbiota with immunotherapy response; Gut microbiome analysis in GI cancer patients receiving anti–PD-1/PD-L1 treatment; 16S rRNA taxonomy survey of fecal samples before and during treatment; Shotgun metagenomics	Anti–PD-1/PD-L1	Prevotella/Bacteroides ratio elevation in responders; higher abundance of specific bacteria (e.g., *Prevotella*, *Ruminococcaceae*, *Lachnospiraceae*) in a responder subgroup; differential abundance of certain metabolic pathways in patients showing different responses; SCFA-producing bacteria positively associated with treatment response; identified bacterial taxa predictive of patient stratification	(Peng et al., 2020)
Advanced thoracic carcinoma	42 patients	Study Association of baseline gut commensal microbes with treatment efficacy; Analysis of gut microbiome in thoracic carcinoma patients receiving anti-PD-1 treatment; Baseline and time-serial stool sample analysis using 16S rRNA gene sequencing of stool samples; assessment of tumor responses, progression-free survival (PFS), and overall survival	Anti-PD-1	5 families (*Kkermansiaceae*, *Enterococcaceae*, *Enterobacteriaceae*, *Carnobacteriaceae* and *Clostridiales* Family XI) bacterial families higher in responders; Consortium of the five families better stratified clinical responses; Higher abundance of microbes associated with prolonged PFS; Abundance of consortium an independent predictor of immunotherapy response	(Yin et al., 2021)
Metastatic melanoma (anti-PD-1 refractory)	10 patients	Study modulation of gut microbiota to influence tumor response; Phase 1 trial assessing safety/feasibility of FMT in patients with anti–PD-1–refractory metastatic melanoma; assessment of safety, feasibility, and clinical responses	Anti–PD-1 reinduction	Clinical responses observed in 3 patients (2 partial responses, 1 complete response); FMT associated with favorable immune and gene expression changes in gut and tumor; favorable changes in immune cell infiltrates and gene expression in gut and tumor microenvironment post-treatment	(Baruch et al., 2021)
Advanced melanoma (PD-1 refractory)	15 patients	Phase 1 clinical trial evaluating safety/efficacy of responder FMT + anti-PD-1 in patients with anti–PD-1–refractory metastatic melanoma	Anti-PD-1	A combination of fecal microbiota transplantation (FMT) and anti–PD-1 was well tolerated in patients with PD-1–refractory melanoma; The combination provided clinical benefit in 6 of 15 patients; Responders exhibited increased abundance of taxa previously associated with anti–PD-1 response and they had an increased CD8+ T cell activation and decreased interleukin-8–expressing myeloid cells.	(Davar et al., 2021)
Melanoma	64 melanoma patients and 10 healthy subjects	Baseline gut microbiome composition and its association with anti-PD-1 response; 16S rRNA sequencing, questionnaire analysis	Anti-PD-1	In responder patients (those who responded to anti-PD-1 therapy), the ratio of *Bacteroidota* to *Firmicutes* bacteria was higher and microbial richness was decreased compared to non-responders; Higher abundance of *Prevotella copri* and *Bacteroides uniformis* bacteria was associated with response to therapy; Non-responders had higher levels of *Faecalibacterium prausnitzii, Desulfovibrio intestinalis*, and some unclassified *Firmicutes* bacteria; Dietary patterns including higher plant, dairy, and fat consumption were associated with better therapeutic response; Gastrointestinal tract functioning was also significantly associated with effects of the anti-PD-1 therapy in melanoma patients.	(Pietrzak et al., 2022)
Various solid tumors (melanoma, NSCLC, renal cell cancer, hepatocellular carcinoma)	27	Collection and analysis of stool samples from patients receiving anti–PD-1 and chemotherapy; Fecal metagenomic sequencing; comparison of microbiota diversity and composition	Anti–PD-1 + Chemo	At baseline, genera like *Parabacteroides*, *Clostridia bacterium* UC5.1_2F7, and *Bifidobacterium dentium* were enriched in responder (R) group, while *Bacteroides dorei* and *Nocardia* species were enriched in non-responder (NR) group; At 6 weeks, beta diversity was significantly different between R and NR groups. Genera like *Alipes, Parabacteroides, Phascolarctobacterium, Collinsella, Ruminiclostridium, Porphyromonas, Butyricimonas* and *Fibrobacteraceae* were more abundant in R group. Genera like *Enterococcus, Lachnoclostridium, Hungatella, Bilophila, Pseudonocardiaceae and Beijerinckiaceae* were more abundant in NR group; Abundance of *Weissella* increased significantly at 6 weeks in R group, while *Fusobacterium* and *Anaerotruncus* increased at 12 weeks in NR group; *Bacteroidetes*, especially *Bacteroides*, were enriched in non-adverse events (NAE) group. Firmicutes like *Faecalibacterium prausnitzii*, *Bacteroides fragilis*, *Ruminococcus lactaris* were enriched in adverse events (AE) group.	(Wu et al., 2022)
Hepatocellular Carcinoma (HCC) with Cirrhosis	11 patients	Study gut microbiota profiles and immunotherapy-induced microbial composition changes as potential biomarkers for clinical outcomes in cirrhotic patients with hepatocellular carcinoma. Gut microbiota profiling, fecal calprotectin, serum zonulin-1, lipopolysaccharide binding protein (LBP), and PD-L1 levels were measured at baseline and during treatment; Patients were categorized into disease control (DC) group (responders) and non-responders; Relative abundance of bacterial taxa was compared between groups.	Anti CTLA-4 and/or anti PD-1	Lower fecal calprotectin and PD-L1 levels at baseline were associated with disease control; Increased *Akkermansia* and decreased *Enterobacteriaceae* abundance was associated with disease control; Fecal calprotectin levels changed in opposite direction to *Akkermansia/Enterobacteriaceae* ratio and alpha diversity during treatment; *Akkermansia* and *Bifidobacterium* abundance associated with other taxa during treatment; Favorable baseline microbiome and lower inflammation associated with treatment response; Intestinal environment changed dynamically during immunotherapy	(Ponziani et al., 2022)
Advanced Cutaneous Melanoma	165 patients plus 147 from previous studies	Association of gut microbiome with ICI response; Shotgun metagenomic sequencing of stool samples collected before ICI initiation from five observational cohorts recruiting ICI-naive patients	ICIs (type not specified)	Microbiome-response associations are cohort-dependent; No consistent microbial biomarker identified; Role of microbiome in ICI response more complex than a simple presence/absence of species. *Bifidobacterium pseudocatenulatum*, *Roseburia* spp. and *Akkermansia muciniphila*, associated with responders was identified, but no single species could be regarded as a fully consistent biomarker across studies. Few microbial biomarkers were consistently associated with response across all datasets. *Roseburia* species were increased in responders. *Bacteroides clarus* was increased in non-responders.	(Lee et al., 2022)
Advanced Hepatobiliary Cancers	65 patients	Analysis of gut microbiome in patients receiving anti-PD-1 treatment to study Association with anti-PD-1 response; Metagenomic sequencing of stool samples; identification of differentially enriched taxa	Anti-PD-1 treatment	The gut microbiome composition is associated with response to anti-PD-1 immunotherapy in patients with advanced hepatobiliary cancers; higher abundance of certain bacteria (e.g. *Lachnospiraceae bacterium*-GAM79, *Alistipes* sp *Marseille*-P5997) was associated with better clinical benefit, progression-free survival, and overall survival; higher abundance of other bacteria (e.g. *Veillonellaceae, Ruminococcus calidus*) was associated with lack of clinical benefit and worse survival outcomes; Microbiome diversity and composition were also correlated with adverse events from immunotherapy, implying the microbiome may impact toxicity as well as efficacy.	(Mao et al., 2021)
Non-Small-Cell Lung Cancer (NSCLC)	338 patients	shotgun-metagenomics-based microbiome profiling on stool samples from patients with advanced NSCLC treated with immune checkpoint inhibitors (ICIs); Assessed the association between baseline fecal levels of Akkermansia muciniphila (Akk) and objective response rates and overall survival.	second- or third-line anti-PD-1	Higher baseline Akk levels were associated with increased response rates and overall survival, independent of PD-L1 expression, antibiotics, and performance status; Intestinal Akk was accompanied by higher levels of other commensal bacteria like *Eubacterium hallii* and *Bifidobacterium adolescentis* in some patients; This also coincided with a more inflamed tumor microenvironment in a subset of patients; Antibiotic use was associated lower Akk levels below 4.8% but increased levels of Akk and Clostridium, both of which were associated with resistance to ICI	(Derosa et al., 2022)
Advanced Melanoma	97 patients	Assessment of impact of *H. pylori* on outcomes and microbiome composition; *H. pylori* serology and fecal microbiome profiling with metagenomics sequencing	81% of patients treated with anti-PD-1 12% Anti PD-1 + anti-CTLA-4.	22% of the 97 advanced melanoma patients treated with immunotherapy were *Helicobacter pylori* positive (*H. pylori* Pos); *H. pylori* Pos patients had significantly shorter overall survival, lower objective response rates, and decreased progression-free survival compared to *H. pylori* negative patients; Specific taxa abundances differed between groups: *Eubacterium ventriosum*, *Mediterraneibacter torques*, and *Dorea formicigenerans* were increased in *H. pylori* Pos group, while *Alistipes finegoldii, Hungatella hathewayi and Blautia producta* were increased in *H. pylori* Neg group. In a validation cohort of NSCLC patients, diversity indices were similar between *H. pylori* groups, but *Bacteroides xylanisolvens* was increased in *H. pylori* Neg patients.	(Tonneau et al., 2022)
Advanced gastric cancer	77 patients	Association of *H. pylori* with anti-PD-1 response; Retrospective analysis of H. pylori association with anti-PD-1 antibody effectiveness; Comparison of outcomes between *H. pylori* pos/neg groups; Analysis of disease control rate (DCR), overall survival (OS), and progression-free survival (PFS) in relation to *H. pylori* status	Anti-PD-1	*H. pylori* positive patients had higher risk of nonclinical response, shorter OS and PFS; *H. pylori* infection independently associated with PFS	(Che et al., 2022)

Developing microbiome-informed therapeutics to treat cancer still has several challenges. Challenges include the resilience of the gut microbiota which makes achieving a sustainable change in microbiota hard (Bloom et al., 2021). Also, the relationship between specific microbiota species and therapeutic outcomes in cancer patients is not fully understood due to unpredicted and undesigned outcomes of clinical trials (Matson et al., 2018). To further compound the difficulty of utilizing microbiome elements to enhance therapies, the is a body of evidence showing that microbiome in older patients commonly lead to detrimental clinical outcomes. The decreased diversity and loss of beneficial species in the microbiomes of older hosts results in detrimental effects such as decreased vaccine efficacy, chronic inflammation, and chronic illness (Bosco and Noti, 2021; Ghosh et al., 2022). Given the extensive relationship between the microbiome and immunotherapy response, it is unsurprising that aging related microbiome changes can effect immunotherapy response, with potential effects being particularly noticeable in ICB therapy (Spakowicz et al., 2021). To make the translation of the microbiome research into microbiome-informed therapeutic interventions in cancer patients we must improve our modeling capabilities for elucidating underlying tumor-immune-microbiome mechanisms and interactions.

## 3 Cancer modeling

In efforts to best study tumor pathophysiology and therapeutic responses, researchers employ a multitude of *in vivo* and *in vitro* platforms, each with their own benefits and limitations (**Table 2**). Two-dimensional plate-based cell culture and animal-based cancer models utilize almost exclusively different methodologies to examine disease growth and progression.

**Table 2:** Advantages and limitations across 2D and 3D culture systems and animal models.

	2D culture	Animal Models	3D culture (Organoids)
Advantages	▪ Traditional and widely used method ▪ Scalability for high-throughput assays and cost-effective (Ravi et al., 2015) ▪ Easily reproducible and standardized ▪ Simple for basic cancer research, genetic manipulation, genetic pathway analysis, and drug screening ▪ Simple to maintain and observe.	▪ Capture complexity of tumor development in whole organisms ▪ Allow study of human-derived tumors in living systems (xenografts) ▪ Useful for tumor development and drug discovery research ▪ Provide a complete physiological system ▪ Allow for study of tumor-host interactions ▪ Enable investigation of metastasis and angiogenesis ▪ Useful for testing systemic effects of treatments (Garcia et al., 2020) ▪ Provide a holistic view of cancer biology (Yee et al., 2015).	▪ A middle ground between 2D cell culture and animal models, providing a more physiologically relevant environment for studying cancer (Xu et al., 2018) ▪ Amenable to genetic manipulation and drug screening ▪ Allow cultivation of multiple cell types and inclusion of stromal components ▪ Exhibit scalability for high-throughput assays (Taelman et al., 2022) ▪ Recapitulate 3D tissue functionality ▪ Patient-derived organoids allow for personalized medicine approaches ▪ Recapitulate histopathological and molecular diversity of original tumors (Chen et al., 2022)
Limitations	▪ Limited validity due to genetic and phenotypic drift (Torsvik et al., 2014; Yu et al., 2019; Quevedo et al., 2020) ▪ Lack of cell-cell and cell-extracellular matrix interactions (Luca et al., 2013) ▪ Reduced ability to model *in vivo* cancer- associated pathways (Luca et al., 2013). ▪ Not accurately represent *in vivo* tumor heterogeneity (Subia et al., 2015) ▪ Lacks the ability to mimic complex 3D structures and physiological environments found *in vivo* (Ravi et al., 2015).	▪ Expensive and Time-consuming (up to 6 months or longer for xenografts) (Abdolahi et al., 2022) ▪ Limited reproducibility ▪ May not support all tumor types (Abdolahi et al., 2022) ▪ Differences in tumor development, microbiome, and immune system from humans (Mestas and Hughes, 2004; Dawson et al., 2009) ▪ Ethical concerns and regulatory challenges ▪ Species-specific differences may limit translatability to humans ▪ Genetic and environmental variability can affect results ▪ May not fully recapitulate human tumor microenvironment ▪ Not always representative of human biology (Garcia et al., 2020).	▪ More complex and expensive than 2D cultures ▪ May not fully capture tumor microenvironment complexity ▪ Standardization and reproducibility can be challenging ▪ Lack of systemic components (immune system, vasculature) ▪ May not fully replicate all aspects of *in vivo* tumor growth and metastasis.

Conventional *in vitro* platforms include numerous tumor cell lines used in 2D formats for genetic pathway analysis and drug screening. While these models contribute important findings to the field of cancer biology and produce high throughput results and easily replicable, cell lines can have limited validity. With continual passages, genetic and phenotypic morphology can drift from the original tumor composition (Torsvik et al., 2014; Yu et al., 2019; Quevedo et al., 2020). Additionally, lack of cell-cell and cell-extracellular matrix contact in traditional 2D cultures reduces cell capacity to faithfully model *in vivo* cancer-associated pathways and interactions (Luca et al., 2013).

*In vivo* cancer models include genetically engineered animals to mimic human tumors, patient and cell line derived tumor xenografts, and spontaneous tumor development in veterinary species. While these models more readily capture the complexity of tumor development in the whole organism, they can be limited by their reproducibility, cost, and construction time. Murine xenograft models, which allow for the study of human derived tumor and associated microenvironment in a living system, are expensive, require immunocompromised host animals, can take up to 6 months or longer to produce, and cannot support all tumor types (Abdolahi et al., 2022). More readily available genetically engineered animals, typically mice, serve an important role in tumor development and drug discovery research. However, their predictive value in translational research can be limited by the lack of etiologic similarity in tumor development and key microbiome and immune system structure differences from humans (Mestas and Hughes, 2004; Dawson et al., 2009). Spontaneous, naturally occurring tumor animal models, particularly in nonhuman primates, allow for effective translational study of tumor development and tumor, immune, microbiome interaction (Deycmar et al., 2023). Yet, these animals also incur significant expense, can have limited throughput, and not all human tumors are represented in other species. To bridge the limitations between the conventional 2D tumor cell culture models and *in vivo* models, tumor organoids are quickly gaining popularity.

### 3.1 Bioengineered tumor organoids in cancer modeling

**Figure 1:**
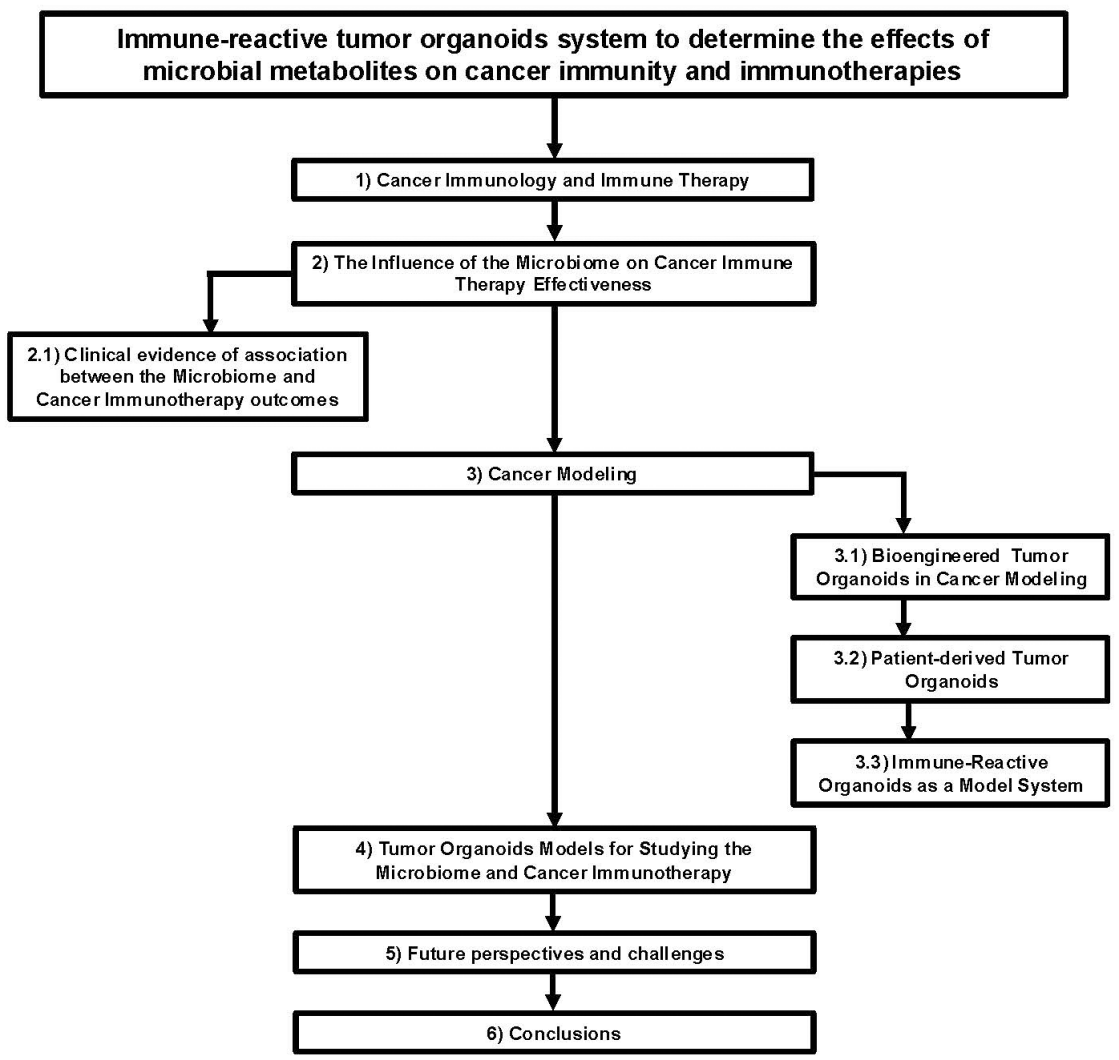
Flow chart diagram describing the application of immune-reactive tumor organoids to study the effects of microbial metabolites on cancer immunity and immunotherapies.

Cancer organoid models provide a reliable platform that bridges the gap between the 2D cancer cell lines and animal models. The tumor microenvironment (TME) is described as a heterogeneous and dynamic milieu composed of stromal, cancer, and immune cells surrounded by a dynamic extracellular matrix (ECM) undergoing continuous remodeling that substantially impacts cancer promotion, progression, and metastasis. ECM plays a key role in shaping the cancer treatment response (Galdiero et al., 2018; Arneth, 2019). Through recapitulating the heterogeneity of the tumor microenvironment cancer organoids provide a relevant pre-clinical model to study cancer pathophysiology. The basic technology behind tissue organoid models utilizes extracellular matrices to support self-organizing of different cell types in a 3D culture and create a more physiologically relevant tissue model. With proper use, these models can better simulate both overall morphology and cell proliferation, differentiation, and migration (Fair et al., 2020; Gjorevski et al., 2022; Mead et al., 2022). Particularly in the context of cancer, bioengineered cancer organoid consist of cancer cells embedded with stromal cells such as the cancer associated fibroblasts (CAFs) encapsulated in specialized ECM based support (**Figure 1**). These cancer cells can be obtained from established cell lines such as shown in the study by Oz et al., where Hep3B, Huh7, and HepG2 cell lines were encapsulated in Matrigel to produce hepatocellular carcinoma (HCC)-like organoids (Oz et al., 2021). Alternatively, unsorted tumor and stromal cells derived from the patient’s tumor are enclosed within specialized hydrogel or ECM to facilitate the formation of organoids (Nagle et al., 2018; Mazzocchi et al., 2018; Votanopoulos et al., 2019a; Forsythe et al., 2021). Organoids derived from patient samples preserve the heterogeneity of the original tumor, offering a more accurate *in vitro* representation of the tissue. Various natural and synthetic ECM options are available for organoid model generation. These include natural ECM components like Collagen type I, Matrigel, decellularized tissues (Giobbe et al., 2019), synthetic hydrogels and recombinant proteins. These materials have been reported to support the growth of organoid models, providing diverse environments for studying cancer within a controlled setting (Kozlowski et al., 2021). The organoids’ 3D architecture was found to alter protein expression and chemosensitivity compared to 2D cultures. Unlike 2D cancer lines, the Cancer organoid model comes with many advantages in cancer research and drug development. They preserve the heterogeneity of the parent tissues and allow the study of drug response and resistance mechanisms. Also, it is a convenient platform to understand the cell’s crosstalk and the role of the stromal compartment in modulating cancer progression and shaping drug response. These models grow relatively quickly, facilitating high-throughput screening and personalized medicine approaches Furthermore, cancer organoids are more cost-effective and human-relevant than animal studies, providing an edge in genetic manipulation experiments (**Table 2**). Several 3D tumor models were reported starting from the multicellular spheroids that were generated to provide the heterogenous cancer microenvironment. Multicellular spheroids are scaffold-free 3D models that are easy to generate and used for cancer research studies because of their simple production through hanging drop or rotatory methods. They are used for several applications including drug screening. A study by Kim et al. generated HCC multicellular spheroids through the co-culture of HEPG2 and insulin-secreting cell line (RIN-5F). They reported higher albumin secretion, which reflected an augmented cell functionality in the 3D heterogeneous culture condition (Kim et al., 2012). In another study Hwang’s team generated pancreatic ductal adenocarcinoma organoids through the co-culture of PANC-1 tumor spheroids with pancreatic stellate cells encapsulated in a collagen matrix. This model enabled them to study ECM remodeling in the context of EMT and anti-invasiveness treatment efficacy (Hwang et al., 2019). In another example, pancreatic cancer organoids were generated from the co-culture of S2-013 cell line, HUVEC, and mesenchymal stem cells, all encapsulated in Matrigel and supplemented with cancer organoid medium to be used later for drug screening (Tanaka et al., 2022). Gastric cancer organoids were also generated from gastric cancer tissues after being digested and then embedded in Matrigel for anti-cancer therapeutics screening (Steele et al., 2019). Through the same approach, cancer organoid models were reported to be engineered for breast cancer (Berndt-Paetz et al., 2023) and bladder cancer. Significant research efforts have focused on enhancing the biological relevance of cancer organoid models by addressing reported limitations such as insufficient heterogeneity, absence of vasculature, suboptimal ECM scaffolds, and lacking immune components. Additional elements such as microbes and their metabolites can be added to create a biomimetic tumor microenvironment (Kadosh et al., 2020; Wan et al., 2021; Shelkey et al., 2022; Xiao et al., 2022). As such, the tumor organoids better mimic *in vivo* characteristics and can be applied as unique models in biomedical research and clinical practice applications precision medicine (Myungjin Lee et al., 2013; Imamura et al., 2015; Riedl et al., 2017; Dzobo et al., 2018; Melissaridou et al., 2019).

### 3.2 Patient-derived tumor organoids

Precision medicine dramatically changes the clinical approach for disease prevention, diagnosis, and treatment from a one size fits all approach to using a patient’s unique genetic, molecular, immune and cellular profile to guide clinical decisions. As this methodology gains traction in cancer therapeutic research, patient biopsy derived tumor organoids are critical for understanding inter-patient and intra-patient differences in tumorigenesis and treatment sensitivity (Xing et al., 2021; Guan and Huang, 2022). Tumor biopsies, obtained through surgical resection of primary or metastatic tumor sites, can be used to produce patient specific tumor organoids. These preclinical models more accurately capture disease genomic complexity compared to traditional 2D cell line models (Xia et al., 2019). Additionally, with increasing numbers of patient-derived tumor organoid studies, living biobanks of tumors are being established to aid in the discovery of subtype heterogeneity, novel drug targets, and therapeutic screening (Fujii et al., 2016; Yan et al., 2018; Guan and Huang, 2022).

Recent publications from Forsythe et al., 2020, 2022, and 2023, and Votanopoulos et al., 2019a, highlight the application of tumor organoids from sarcoma, peritoneal mesothelioma, colorectal, and appendiceal cancers for chemotherapeutic efficacy screening on an individual patient basis (Votanopoulos et al., 2019a; Forsythe et al., 2020; Forsythe et al., 2022a; Forsythe et al., 2023). These studies underscored the improved chemo-response modeling of tumor organoids compared to 2D cell lines when comparing chemotherapeutic responses (Forsythe et al., 2020). Beyond their usefulness to model patient-specific chemo-response, tumor organoids created from different metastatic sites within the same patient showed differential therapeutic response, demonstrating the ability to model intra-patient lesion-specific chemo-response and the underlying disease clonality (Forsythe et al., 2023).

### 3.3 Immune-reactive organoids as a model system

By integrating patient matched immune cells such as T cells, macrophages, and dendritic cells, tumor-immune cell organoids have emerged as an important platform to study tumor-immune interactions critical to tumor progression and therapeutic response. As previously mentioned above, numerous studies have utilized autologous immune enhanced patient derived tumor organoids in efforts to predict clinical immune checkpoint inhibitor success (Votanopoulos et al., 2019a; Forsythe et al., 2020; Forsythe et al., 2022a; Forsythe et al., 2023). On a more mechanistic level, tumor-immune cell or immune-reactive organoids serve the purpose to study immune cell infiltration into the tumor mass (Tsai et al., 2018). This is essential for investigating the spatial distribution of immune cells within the TME and the immuno-biologic reactions in the tumor microenvironment.

**Figure 2:**
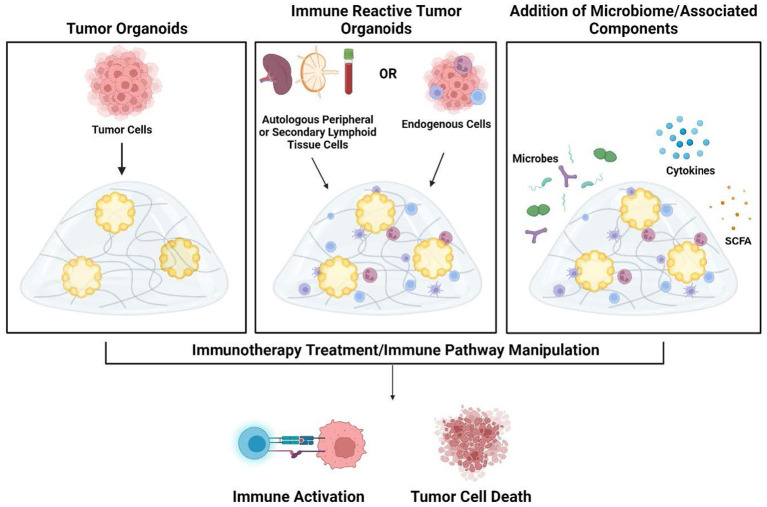
Development of tumor organoids to test immune response pathways, tumor cytotoxicity, and microbiome impact. Organoids are created by encapsulation of dissociated tumor cells in extracellular matrix hydrogels (left panel). To create immune-reactive organoids, immune cells can be isolated from either autologous peripheral blood/secondary lymphoid organs (lymph node or spleen) or isolated from the tumor. Immune cells can then be directly incorporated into the 3D matrix (middle panel). As an additional component, microbes or their metabolites and products such as cytokines and short-chain fatty acids can also be added to the organoid cultures (right panel). Once culture systems are established, testing such as the addition of immunotherapy treatments can be performed to analyze subsequent immune activation and tumor cell death within the organoids. Figure created in BioRender.

Immune-reactive organoid platforms can be established by incorporating immune components through one of two primary strategies: 1) retaining endogenous immune cells that are intrinsically present in parental tissue, and 2) co-culturing autologous immune cells with tissue-matched tumor organoids (**Figure 2**).

#### 3.3.1 Retention of endogenous immune cells from parental tissue strategy

In this strategy, an unsorted cell population, including endogenous immune cells from normal or cancerous tissue, was mixed and encapsulated into various ECM or hydrogels to create tumor immune organoids.

In this innovative approach of immune reactive organoid culture system, efforts are made to maintain an environment conducive to the survival and proliferation of both the tumor cells and resident immune cell population, specifically macrophages and natural killer cells. Several non-tumor organoids have demonstrated the success of these models. A recent study derived mouse adipose organoids via enzymatic digestion of C57BL/6 visceral fat tissue, grown in ultra-low attachment plates to form spheroids. This culture system was able to retain resident macrophage cells, which are critical participants in lipid metabolism. The resulting immune-enhanced model enabled studying innate immune-adipocyte interplay (Taylor et al., 2020). In another work, Kue et al. reported a lung organoid model cultured at an air-liquid interface that retained endogenous lung tissue-resident immune subsets including T-cells, B-cells, natural killers, and myeloid cells (Choi et al., 2023). This immune-enhanced lung organoid provided a significant advance in modeling tissue-resident immunity through an integrated immune-reactive organoid and was used to study T cell activation and responses to SARS-CoV-2 virus exposure. Wan et al., described the use of this organoid culture approach in generating high-grade serous ovarian cancer (HGSC) immune reactive organoids. They used this model to evaluate the efficacy of simultaneous use of PD-1 and PD-L1 Immune Checkpoint Blockades in HGSC (Wan et al., 2021). In another study, Neal et al., developed patient derived tumor organoids with endogenous immune and stromal elements for *in vitro* immunotherapy modeling (Neal et al., 2018). They found that the inclusion of the native tumor infiltrating lymphocyte population allowed for functional activation, expansion, and cytotoxic response to PD-1/PD-L1 checkpoint blockade therapy.

A key advantage of strategies retaining endogenous immune cells is the preservation of physiologic immune composition diversity and heterogeneity reflective of parental tissue. This better captures the complex dynamics of immune cell interplay with other organoid components versus simplified co-culture approaches. However, maintaining the reproducibility of heterogeneous models with consistent phenotypic stability remains an ongoing challenge.

#### 3.3.2 Co-culture with autologous immune cell types

The most common coculture approaches for the generation of immune-reactive organoids involve coculturing tissue-derived cells with autologous immune cells, including those derived from peripheral blood or secondary lymphoid tissues such as lymph nodes and spleen (Votanopoulos et al., 2019b; Forsythe et al., 2022b; Shelkey et al., 2022).

Shelkey et al. developed an immune-reactive organoid system that included murine colon adenocarcinoma and breast cancer cell lines. Tumor organoids were cocultured with T-lymphocytes derived from murine lymph node tissue. These tumor immune organoids were fabricated by encapsulating the tumor and immune cells in a modified collagen based hydrogel (Shelkey et al., 2021). In another study, they reported generating a similar immune reactive system via coculture of the 4T1 TNBC murine cell line and matched splenocytes. Both models were used as platforms for testing the influence of bacterial metabolites on the efficacy of checkpoint inhibitors PD-1 and CTLA-4. They reported a beneficial effect of the bacterial metabolite on immune cell viability and potency. They also concluded there was a synergistic effect of the bacterial metabolite on the immunotherapy regimen (Shelkey et al., 2022).

Immune reactive organoid models are also generated from patient-derived tumor samples to model the tumor microenvironment and predict immunotherapy response. A patient-specific, immune-enhanced organoid platform for melanoma was developed by co-culturing either lymph node cells or peripheral blood mononuclear cells into matched patient tumor-derived organoids (Votanopoulos et al., 2019b). Organoid response to immunotherapy drugs like nivolumab and pembrolizumab showed 85% predictive accuracy compared to actual patient clinical outcomes. This study advanced personalized immune therapy regimens using patient-derived organoids. Similar investigations into tumor immunobiology utilized a patient-derived organoid model of gastric cancer co-cultured with PBMC-derived immune cells. Here, Chakrabarti et al., 2021 found that HER2 regulates PD-L1 expression in gastric cancer to drive tumor-immune cell evasion (Chakrabarti et al., 2021). These findings support further research into combinatorial therapy for gastric cancer, as well as the use of organoid/immune co-cultures to screen for targeted therapeutic approaches.

In the same vein, immune cells can be sourced from the tumor sample itself via coculture of tumor-infiltrating lymphocytes (TILs). TILs are isolated from tumor samples, expanded *ex vivo*, then reintroduced to tumor cells to potentially enhance anti-tumor cytotoxicity toward specific antigens (Magré et al., 2023). One study reported a co-culture system between patient-derived rectal cancer tumoroids and matched TILs that were isolated, expanded, and reintroduced. This immune reactive system was used to assess the immune response to checkpoint blockade inhibitors. The study showed a restored TIL cytotoxicity and increased PD-1 expression upon treatment with anti-PD-1 antibody (Kong et al., 2018).

The co-culture system permits the pretreatment and genetic modification of immune cells, such as CAR-T cells, to target specific antigens. This aspect is crucial for advancing the mechanisms and methods in immunotherapy (Yu et al., 2021). Furthermore, these systems facilitate research into immune cell role in shaping tumor behavior and drug responses at both the cellular and the more expansive tissue-mimetic organoid levels. A notable example of this approach is the work by Jiang et al., who elucidated the role of macrophages in modulating Gemcitabine resistance in pancreatic adenocarcinoma. They generated an immune-reactive model using a co-culture of patient-derived tumor samples and tumor-derived macrophage cells. This study revealed the critical role of the CCL5-p1-AREG axis feedback loop between macrophages and pancreatic cancer cells (PCCs) in conferring drug resistance (Jiang et al., 2023).

From these examples and numerous others, tumor organoid platforms are increasingly recognized as a novel means to both improve our understanding of immunotherapy mechanisms and drive therapeutic progress.

## 4 Tumor organoids models for studying the microbiome and cancer immunotherapy

Recent developments in biomaterials have resulted in more physiologically accurate culture methods that can be used to study complex human systems (Rossi et al., 2018). Specifically, advances in immune population and microbiome *ex vivo* models have allowed for composite models to examine interactions between the two systems (**Figure 1**). Organoids have been used to model many aspects of the microbiome and immune environment including different bacterial species, viral infections, and effects on various cellular niches (Min et al., 2020). Anti-PD-1 and anti-CTLA-4 have been tested in microfluidic devices to model efficacy (Aref et al., 2018). Organoids and spheroid culture have also demonstrated their use as a model for studying ICI efficacy (Jenkins et al., 2018; Neal et al., 2018). Microbiome derived factors and viable immune responses can therefore be combined to create a model that can demonstrate the interplay between microbiome derived metabolites and ICI in an organoid system (Shelkey et al., 2022). Chip systems have even been constructed that incorporate microbiota compartments to produce immunomodulatory effects on the tissue present with specific species causing identifiable inflammatory responses through factors like metabolite production and reactive oxygen species (De Gregorio et al., 2022). It is even possible to modulate the microbiome in chip-based systems to cause inflammation and bacterial outgrowth, which can then be monitored in ways that incompatible with animal models (Kim et al., 2016). The high throughput nature of *ex vivo* culture makes it ideal for conducting large scale studies that work to isolate individual components underpinning the mechanisms of action for complex systems (Rae et al., 2021). Further advances in advanced cell culture models will continue to contribute to understanding immunotherapy-microbiome interactions.

## 5 Future perspectives and challenges

*Ex vivo* culture of cells and tissue constructs is continuously advancing with applications in precision medicine, immune modeling, and organ system replication. Organoids have proven to be particularly effective at predicting cancer patient response to therapeutic treatment (Nagle et al., 2018; Mazzocchi et al., 2018; Votanopoulos et al., 2019a; Forsythe et al., 2021). Advances in microfluidic production have allowed multiple organ systems to be integrated in one construct to demonstrate how all of the different systems interact (Skardal et al., 2017; Ingber, 2022). These systems are also significantly better for continuous monitoring of cell populations of interest (Kim et al., 2016). Microbiome modulation and analysis in correlation with cancer immune therapy and cancer progression has been investigated in several clinical studies to evaluate the positive outcomes and drawbacks. These studies also aim to estimate the potential of antibiotics that affect gut microbiome composition and the subsequent influence on either cancer promotion or response to therapeutics. There have previously been difficulties in producing *ex vivo* culture models that are exposed to live bacterial populations, with tissue and live bacterial interactions being limited to animal models. Some researchers compensated by focusing on bacterial metabolites that could be used in aseptic culture; however, intestine-on-a-chip models have now been produced that are able to sustain equivalent microbiomes in culture with intestinal epithelium. These models replicate oxygen gradients, intestinal barriers, and can be utilized going forward for the controlled testing of therapeutics (Jalili-Firoozinezhad et al., 2019; Shin et al., 2019). Different versions of the gut-on-a-chip model can even model peristaltic movements while maintaining a normal microbial population (Kim et al., 2012). With the continuous advances in cell culture technology producing models that were previously impossible, it is likely that more *ex vivo* models better able to reproduce the complicated cancer immunotherapy-microbiome interactions will be produced. These models can more easily be leveraged for high-throughput studies both for mechanistic investigation and predictive precision medicine, resulting in better patient care. These results will continue to supplement and corroborate the ongoing clinical trials that aim to elucidate the interactions between the microbiome and cancer immunotherapy.

## 6 Conclusion

With several drugs in the clinical pipeline and more clinical trials in progress, immunotherapies are a promising therapeutic for a wide array of tumor types (Cloughesy et al., 2019; Ni et al., 2021; Rothschild et al., 2021; Vignali et al., 2022; Garon et al., 2023; Huang et al., 2023; Boesen et al., 2024). To continue to grow the list of available immunotherapies, advanced testing platforms are necessary. Tumor organoids, particularly those derived from human primary specimens, are an increasingly important platform to personalize current therapies and model various tumor microenvironment interactions to accelerate novel drug development. In particular, the capacity to co-culture microbiome, immune cells, and tumor organoids provides critical insight into complex interplays which regulate immunotherapy responses. As we highlight in this review, these dynamic mechanisms cannot be accurately modeled in traditional 2D culture systems, and animal models fail to provide fully translational findings. As microfabrication technologies continue to evolve and improve the organoid systems, these *ex vivo* assays are a crucial tool to innovate immunotherapy treatment strategies through understanding of the microbiome-immune-tumor interactions and improve patient outcomes.
